# Identifying Stuttering in Arabic Speakers Who Stutter: Development of a Non-word Repetition Task and Preliminary Results

**DOI:** 10.3389/fped.2022.750126

**Published:** 2022-03-11

**Authors:** Roaa Alsulaiman, John Harris, Sarah Bamaas, Peter Howell

**Affiliations:** ^1^Division of Psychology and Language Sciences, University College London, London, United Kingdom; ^2^Speech and Language Pathology Division, Jeddah Institute for Speech and Hearing, Jeddah, Saudi Arabia

**Keywords:** fluency, stuttering, screening, Arabic, speech disfluency, word-finding, non-word, diversity

## Abstract

Stuttering and other conditions that affect speech fluency need to be identified at an early age in order that effective interventions can be given before the problems becomes chronic. This applies in countries where several languages are spoken including those in which English and Arabic are both widely used which calls for assessment procedures that work across these languages. The ‘universal' non-word repetition task (UNWR) has been established as an effective screening tool for discriminating between children who stutter (CWS) and children with word-finding difficulty for a number of languages. However, the UNWR does not apply to languages such as Arabic and Spanish. The present study aimed to: (1) introduce an Arabic English NWR (AEN_NWR); which was developed based on the same phonologically informed approach used with UNWR; (2) present preliminary non-word repetition data from Arabic-speaking CWS and adults who stutter (AWS). The AEN_NWR items comprises twenty-seven non-words that meet lexical phonology constraints across Arabic and English. The set of items includes non-words of two, three and four syllables in length. Preliminary non-word repetition data were collected from ten CWS between the ages of 6;5 and 16;7 (*M*_age_ = 12:1) and fourteen AWS between the ages of 19;2 and 31;0 (*M*_age_ = 24). Participants performed the non-word repetition task and provided a sample of spontaneous speech. The spontaneous speech samples were used to estimate %stuttered syllables (%SS). To validate that AEN_NWR performance provides an alternative way of assessing stuttering, a significant correlation was predicted between %SS and AEN_NWR performance. Also, word length should affect repetition accuracy of AEN_NWR. As predicted, there was a significant negative correlation between the AEN_NWR and %SS scores (r (25) = −0.5), *p* < 0.000). Overall, CWS were less accurate in their repetition than AWS at all syllable lengths. The AEN_NWR provides a new assessment tool for detecting stuttering in speaker of Arabic and English. Future studies would benefit from a larger sample of participants, and by testing a population-based sample. These studies would allow further investigation of the AEN_NWR as a screening measure for stuttering in preschool children.

Andrews and Harris (1) reported that the lifetime incidence of stuttering is 1% in their study on stuttering in 1,142 families in the United Kingdom that used children born between May and June 1947 in Newcastle, UK. The study ended when the children were 15 years old, and established that the point prevalence of stuttering up to the age 15 was approximately 4.9%. Yairi and Ambrose (2) confirmed that approximately 5% of pre-school age children exhibit episodes of stuttering. Stuttering and other conditions that affect speech fluency need to be identified at an early age so that effective interventions can be given before the problem becomes chronic (3, 4). This applies in countries where several languages are spoken including those in which English and Arabic are both widely used. In countries of the latter type, some children use both languages, and they may be less fluent in the official language used in schools (e.g. English in the UK) than the one they use in their home (Arabic in this example). Whilst it would be possible to wait for fluency in, for instance, English to develop in school before attempting to identify cases of stuttering, this would delay identification and intervention of children affected by fluency issues. Delaying intervention for some time after a disorder has begun may lead to other effects that result in lower educational achievement, and behavioral and social problems in a child's later life (4). Consequently, a school-based screening procedure has been developed for identifying children with Speech Language Communication Needs (SLCN), including stuttering, for use in reception classes (5). Howell's procedure separates fluent children, those children with word-finding difficulty (WFD), which could arise *inter alia* when children use English as an Additional Language (EAL) and those with SLCN. As validation of the procedure, a spontaneous speech sample was obtained from each child, and analyzed for symptoms of stuttering and WFD. Three speech symptoms were used (part-word repetitions, prolongations and word breaks) to identify stuttering which was quantified as percentage of stuttered syllables out of all syllables spoken (%SS) as in Riley's (6) Stuttering Severity Instrument (SSI). Stuttering was identified when children had rates of stuttering symptoms above a threshold %SS which did not preclude them also exhibiting high rates of WWR (7). Any remaining children with %WWR above threshold were designated as having WFD whilst children below %SS and %WWR thresholds were designated fluent.

School staff and teachers do not have much time to dedicate to SLCN because they are under pressure to deliver national curriculums (4). Consequently, Mirawdeli (8) argued that assessment procedures are needed that are quick and practical to use in schools. A further complication is that large numbers of pre-schoolers who use a wide number of native languages need to be screened. This requires forms of assessment that are independent of language spoken. To this end, Howell et al. (9) developed a “universal” non-word repetition task (UNWR) that is convenient to administer and score and applies to at least 20 languages^1^ spoken in UK schools. Howell et al. (9) showed that the UNWR is an effective screening instrument for discriminating between fluent children, children who stutter (CWS) and children with WFD. As a *non-word* test, UNWR controls for extraneous influences of lexical knowledge, making it a sensitive marker for children's phonological ability. UNWR uses consonants that occur in all languages that the test applies to. To generate UNWR test items, the overlapping phonotactic properties of onsets and codas for legitimate syllables in the selected languages were identified, and rules for concatenating syllables for constructing multisyllabic non-words were applied. Next, exemplars consisting of all strings meeting these constraints were automatically generated, and bespoke dictionaries created and used to exclude candidates when the strings were words that occurred in any of the languages included in UNWR. The test was administered to 96 children from reception classes in five mainstream primary schools in the United Kingdom of which 20.83% used EAL (9). The spontaneous speech samples from the children were assessed for symptoms of stuttering and WFD, and their performance on the UNWR was measured. Stuttering symptoms (measured by %SS) predicted UNWR scores, whereas WFD scores (measured by %WWR) did not. The findings were interpreted as confirming that UNWR scores differentiate stuttering from WFD.

The issue addressed in this paper arose because UNWR does not apply to languages with vastly different phonological structures from languages like English, such as Arabic, Spanish and Mandarin. This paper attempted to fill one of these gaps by providing a new NWR task for screening Arabic and English children, the Arabic English NWR (AEN_NWR). AEN_NWR is based on the same phonologically-informed approach used with UNWR. The test items were constructed so that they accord with various constraints on lexical phonology common to Arabic and English. This quick and easy-to-administer test includes stimuli that vary in syllable structure, consonant age of acquisition, lexical effects and stress.

A brief review of the literature on NWR as a potential behavioral clinical marker for identifying language disorders is presented next. This literature has focused on non-word repetition and aspects of phonological performance in CWS as well as children with other language disorders such as specific language impairment (now referred to as developmental language disorder, DLD) and dyslexia. NWR can potentially inform work on how phonology pertains to speech fluency. Following this review, a description of Arabic phonology specifying areas that were involved in the generation of Arabic and English non-words is presented.

## Non-word Repetition as a Language Assessment Tool

The ability to repeat a novel phonological sequence is a basic language skill that humans possess. Infants under the age of 12 months attend to speech sounds, especially when the sounds are spoken to them by adults (10). Infants can spontaneously imitate the words of others and by the time they are 2 years old they repeat non-word when requested (11). Children also repeat “non-words” spontaneously when they mimic real words spoken to them by adults (12). Although non-word repetition (NWR) tasks appear simple, they rely on the following cognitive processes. First, the person must process the acoustic signal, extract phonemes and match the signal with phonological representations in memory. Then, the person must plan the articulatory movements for achieving production of the non-word and execute this plan as their response (13). Correlations between phonological working memory and non-word repetition were first examined by Gathercole and Baddeley (14). Performance on non-word tasks has been examined in children with several language disorders such as dyslexia (15) and specific language impairment (16). Performance on NWR tasks presents a challenge for children with these language disorders. For instance, Snowling's (15) study that examined NWR non-word abilities in children with dyslexia tested two groups of children: 22 typical readers and 20 dyslexic children whose age ranged between 7 and 17 years old. The children repeated 30 non-words that were two, three or four syllables long. non-words posed more difficulty for dyslexic children than for the control group with dyslexic children making more repetition errors. Significant differences between the groups were found when four syllable non-words were repeated. The finding was interpreted as indicating a phonological deficit in dyslexic children. Subsequent studies [e.g., (17)] agree that dyslexia should be considered a phonological deficit indicating language weakness rather than impaired low-level auditory difficulties (18).

## On the Relationship Between NWR and the Phonological Loop

**Figure 1:**
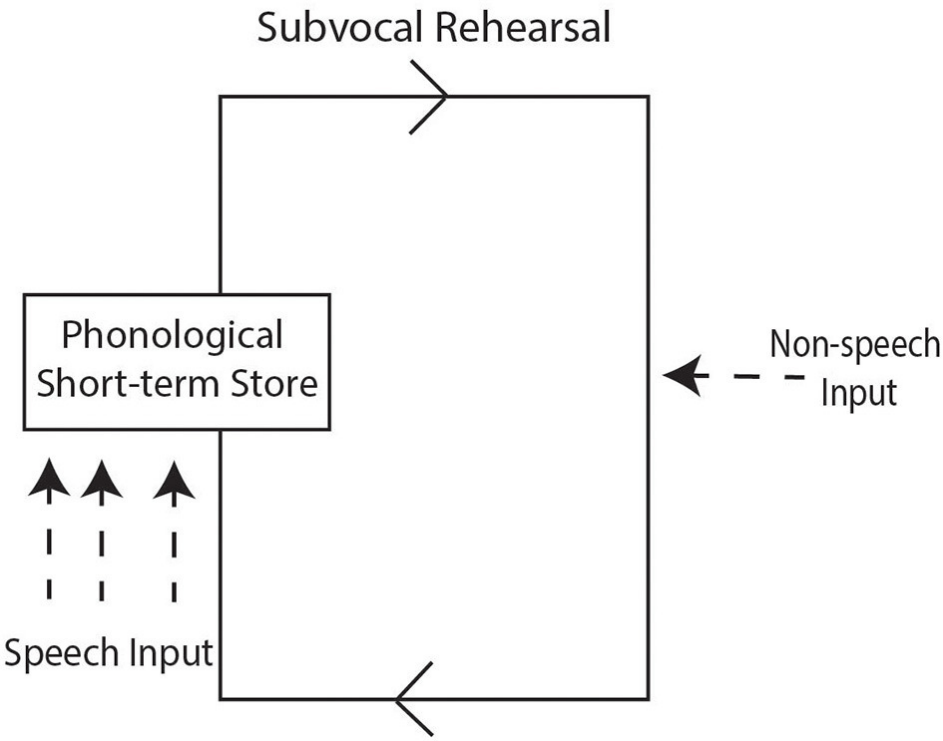
Schematic representation of the phonological loop model based on Baddeley (21) adapted from Gathercole (22), See text for further detail.

Before turning to studies on NWR and stuttering, details are given about phonological memory to illustrate its role in repetition of non-words or unfamiliar words. The phonological loop is part of working memory (WM), which is a cognitive system that temporarily holds and manipulates information whilst people perform tasks such as comprehension and learning (19). The WM-model of Baddeley and Hitch (20) proposed three major components: (1) the central executive system, which is the supervisory controlling system that is aided by the other two components; (2) The visuospatial sketchpad, which is concerned with the visuo-spatial memory; that is, it stores and processes information in a visual or spatial form; and (3) the articulatory loop (now referred to as the phonological loop) that is responsible for rehearsing and storing speech-based verbal information. It transforms the verbal stimuli into phonological codes which have the associated acoustic and temporal properties of the stimuli. Matches between the phonological codes and codes that exist in the long-term memory system (i.e., phonemes and words) are sought. The phonological loop can be further divided into two sub-components: the phonological short-term store and the subvocal rehearsal component (21). The phonological short-term store is like an inner ear which holds speech-based information for up to about two seconds. Speech-based information can be maintained by the subvocal articulatory rehearsal component; a process that can be used to enter information into the phonological store. The subvocal rehearsal component is like an inner voice which allows information to be rehearsed, for example, object names that are articulated either overtly or sub-vocally (21). Rehearsal allows a person to remember a telephone number by circulating the phone digits to oneself. Figure 1 provides a graphical representation of the phonological loop model based on Baddeley (21) adapted from Gathercole (22).

We now provide a brief overview of the way the phonological loop operates when repeating non-words. As implied earlier, it has been established that there is a strong relationship between NWR performance and the phonological loop component of WM (23). Repeating non-words requires temporary storage of unfamiliar phonological sequences in the phonological loop; and it is assumed that success when holding the sequences depends on the short-term memory capacity of the phonological loop. The rehearsal component of the phonological loop serially reactivates the unfamiliar phonological sequence stored in the phonological store, where this process does not necessarily involve movements of speech articulators. As long as rehearsal is maintained, the phonological store can hold on to the speech information. Indeed, the process of rehearsal is time-limited; the longer a phonological sequence is, the longer it takes to reactivate the sequence leading to fewer rehearsals in a given time (22).

## Non-word Repetition and Stuttering

Several studies have reported how NWR performance is affected in CWS and adults who stutter (AWS). Hakim and Ratner (24) investigated the performance of eight CWS and eight children who do not stutter (CWNS) on non-word repetition where the children's ages ranged between 4 and 8 years. Children attempted to repeat 40 non-words from Gathercole et al.'s (23) Children's Test of Non-word Repetition (CNRep). The task consisted of 40 non-words of 2-, 3-, 4-, and 5-syllables in length. Results showed that CWS were less accurate at repeating non-words at all syllable-lengths, although statistical differences between participant groups only occurred for the 3-syllable non-words but not the 2-syllable and longer 4- and 5-syllable words. Anderson et al. (25) replicated and extended the findings from Hakim and Ratners's (24)'s work in a sample of younger children (aged 3 to 5 years). The authors argued that examining non-word repetition performance at this young age could provide an opportunity to assess phonological memory during a time of critical language development, relative to school aged children. Anderson et al. (25) administered the CNRep to 12 CWS and a matched control group of 12 CWNS. CWS were significantly less accurate in repeating non-words of two and three syllables. However, no significant differences were found between the groups in their accuracy of repeating the longer non-words (four and five syllable non-words). The findings of this study is partially consistent with results from Hakim and Ratner (24). The authors suggested that the significant differences between the groups even with the two syllable non-words were probably because this is a younger group of children; thus, their performance was not impacted by ceiling effects. On the other hand, the lack of significance effects for the longer non-words was due to the impact of floor effects in both groups.

Subsequently, Anderson and Wagovich (26) examined the relationships between measures of linguistic processing speed and between two aspects of cognition: phonological working memory and attention. Nine CWS and a matched control group of 14 CWNS, aged 3 to 5 years, participated. Gathercole et al.'s (23) CNRep test was again used in this study. Children were asked to repeat 40 non-words, where there were 10 each of 2-, 3-, 4-, and 5-syllable non-words. There were significant differences between the two groups in their accuracy of repeating non-words of two and three syllables. However, the differences between the two groups were not significant when repeating non-words of four and five syllables, although CWS performed worse (i.e., were less accurate in their repetition) with them than were controls. These findings are consistent with Anderson et al.'s (25) study and the lack of significant differences on the longer nonwords could be attributed to floor effects in both groups.

Sasisekaran and Byrd (27) investigated NWR accuracy in 14 CWS and a matched control group of CWNS aged between 8 and 15 years of age. Participants repeated a set of 36 non-words consisting of 12 non-words at each syllable lengths (2-, 3-, 4-, and 7-syllables). CWS were less accurate when producing two-syllable non-words compared with the CWNS. However, differences between CWS and CWNS on accuracy at each syllable length was not reported. The non-words at four-syllables posed most difficulty for children in both groups (i.e. had the lowest percent of correct repetitions). Based on this result, the authors suggested that their findings are consistent with previous studies; confirming that CWS show a trend to perform poorly on NWR tasks. Whilst the studies above focused on English-speaking children, Sugathan and Maruthy (28) explored NWR performance in Kannada-speaking school-aged children. Seventeen CWS and a matched group of CWNS were tested. The non-words consisted of 2-, 3- and 4-syllables, and for each syllable length there were 12 non-words. These were language specific in that they conformed with the phonotactic constraints of the Kannada language. CWS were less accurate in producing the non-words compared to the CWNS at all syllable lengths. Significant differences between the two groups were reported for the mean number of correct non-words; however, whether the differences at each syllable length were significant was not reported.

Howell et al. (9) investigated NWR ability in a group of 96, 4–5-year old monolingual English children and children with EAL, who came from diverse language backgrounds, using the UNWR. The goal was to evaluate the effectiveness of the UNWR in distinguishing between CWS and children with WFD, irrespective of which language they speak. Children with EAL often have to produce phonological sequences in English; a language they are not familiar with. This is similar to what happens in NWR tasks where children are required to repeat a novel sequence of phonemes that does not exist as a word in their first language. Howell et al.'s (9) study faced the methodological problem, common to many NWR tasks, that materials tend to be biased toward the language for which the test was developed. Howell et al.'s (9) “universal” NWR task was developed to apply to various languages spoken in UK schools hence the name “universal” and avoids confounds between language ability in the test language and presence of stuttering symptoms. If a person has WFD but no stuttering symptoms, this should not be evident when repeating non-words, whereas CWS are expected to struggle performing the task (9). UNWR scores were predicted by %SS, but not by WFD [as measured by the percentage of whole word repetition (%WWR)]. This relationship between UNWR and %SS provided empirical evidence that UNWR provides a sensitive measure of stuttering. The authors also attributed this relationship to the fact that both measures (UNWR and %SS) reflect phonological planning, whilst WFD is more of a vocabulary problem rather than an articulation one. The results also showed that monolingual English children and children with EAL did not differ in their performance on UNWR. Thus, accuracy in repeating non-words on the UNWR was not affected across language groups who showed different levels of %WWR. This again highlights that the test eliminated the problem in other NWR tasks that favor the language for which the test was designed. In summary, the UNWR has a strong potential as a screening instrument for language-diverse samples that can separate CWS from CWNS based on their accuracy in repeating non-words.

Whilst the reason for developing the AEN_NWR is to establish a stuttering screening instrument for preschool *children*, we argue that assessing adults is also necessary for the following reasons. First, when planning assessment and intervention for speech disorders, it is recommended that users are aware of weakness in phonological processing in older participants [e.g. adolescents in (29)]. Second, testing participants at older ages on their performance on NWR aids in capturing developmental differences in repetition accuracy (30). In relation to that, the previous studies presented generally showed that CWS were performing at ceiling levels for the shorter nonwords and at floor effect for the longer ones. Consequently, it would be of interest to evaluate whether ceiling and floor effects operate for AWS; and if they do, at which syllable length would AWS and CWS be differentiated. In fact, it is desirable when designing an NWR to have performance around the ceiling and floor to assess performance limits of participants on items that vary in difficulty (25). Thus, in this section, we present a brief overview of studies that examined NWR performance in AWS. These studies have informed our hypothesis on differences between AWS and CWS on accurate repetition of NWR items.

Several researchers have examined NWR abilities in AWS and AWNS. Namasivayam and Van Lieshout (31) had five AWS and five AWNS repeat a set of non-words at two different rates (normal and fast) across three test session (two sessions on the same day, and a third session approximately one week later). AWS and AWNS differed in variables that concern the organizational aspects of speech motor control in terms of: (1) movement stability and (2) the strength of coordination patterns. Thus, the authors interpreted the results as AWS may have limited unique difficulty in the motor leaning of new sound sequences. Smith et al. (32) explored performance on a non-word repetition task in 17 AWS and a matched control group of 17 AWNS. The non-words ranged in length from one to four syllables; these were adapted from the Non-word Repetition Test [NRT; (33)]. Overall, there were no differences between the two groups. In fact, all participants performed at or near ceiling while repeating non-words of 1-,2-, and 3-syllables length. Only for non-words of 4-syllables and 5-syllables, the AWS scored lower than AWNS, although the results were still comparable. These findings could indicate that, unlike children, longer nonwords may be better at revealing differences between AWS and AWNS. Byrd et al. (34) also examined non-word repetition abilities in 14 AWS and a matched control group of 14 AWNS. Participants repeated non-words that consisted of 2-, 3-, 4- and 7-syllables. The two groups repeated the 2-, 3- and 4-syllable length non-words with comparable accuracy. Only for the 7-syllable length non-words, significant differences in accuracy were found between the two groups with AWS being less accurate in their production. Hence, it was suggested that it might be possible that non-words of at least 7 syllables may be needed to distinguish the two groups, whilst non-words at 2–3 syllable lengths are sufficient when to differentiate between CWS and CWNS Byrd et al. (34).

From the review above, it is clear that the available findings, both from children and adults are mixed. In terms of studies on CWS, it appears that there is a general agreement that CWS score lower than CWNS, but this does not hold true for all syllable lengths. Some studies provided information on how CWS and CWNS performed on NWR, overall and at each syllable length. However, other studies provided only general information on the performance of each group. This poses a limitation in comparing results from those studies. With respect to studies on AWS, there are several issues that are worth noting: (1) performance on non-word repetition tasks for AWS has received less attention; (2) Moreover, the methodology of those studies is not consistent. In some of the studies reviewed, NWR performance was part of a large study and there was not sufficient details on performance at each syllable length. Also, a general conclusion is that AWS might struggle repeating long non-words. This could be an indication that stuttering is associated with limited phonological working memory capacity. Thus, because the AEN_NWR is a newly designed task, we employed a sample of CWS and AWS to investigate group differences in performance at non-words that vary in length and phonological complexity.

To wrap up this section, it is worth noting that the NWR tasks employed in most studies were language specific, making them appropriate for English speakers only. Language-specific NWR tests are needed that test cohorts of speakers who use two or more languages (e.g., Arabic and English here) in an unbiased way.

## Arabic Language

Arabic language has different phonological and morphological structures to English and the other Indo-European languages that UNWR applies to. Both English and Arabic use a pulmonic egressive airstream mechanism. This means that all the speech sounds of English and Arabic are produced using air from the lungs that exits the vocal tract (35). The two languages, however, still differ in many respects such as the phonemes that occur in each (this applies to consonants and vowels), stress and syllable constraints. Another aspect where the two languages differ is that the Arabic plural forms include singular, dual (i.e. referral to two objects or two persons) and plural while the English include two forms only; singular and plural. Additionally, in Arabic, stress depends on the syllable weight and it is more predictable than it is in English. The final syllable is stressed in cases where there is a long vowel (CVV) or where there is a word-final consonant cluster (CVCC), including geminate consonants. In other words, syllables with consonant clusters carry the main stress (36), which made it impossible to manipulate stress independent from consonant clustering when creating non-word stimuli. These differences, along with the phonotactic constraints that apply to both languages were examined below to generate the AEN_NWR stimuli. The steps involved in generating AEN_NWR stimuli that incorporate phonotactic constraints shared by English and Arabic to equitably test speakers of English and Arabic are described. The features of Arabic that governed design of AEN_NWR stimuli are described next.

**Figure 2:**
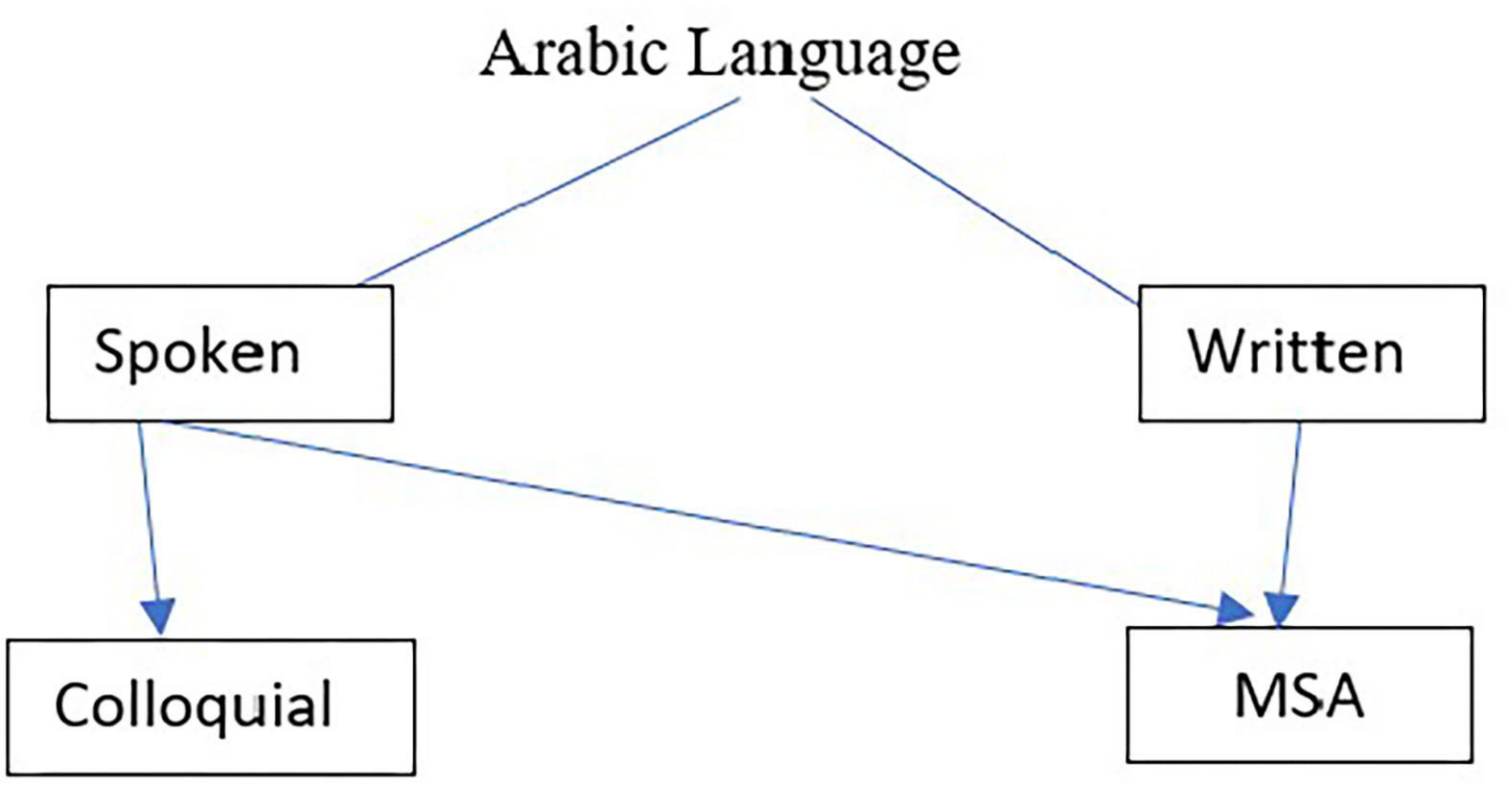
Usage of MSA and CA.

Arabic is the most widely spoken Semitic language in the world and is used by more than 250 million people as their first language in the Middle East. Arabic is considered a Diglossic language (37). There is a Standard form, Modern standard Arabic (MSA) and a large number of regional Colloquial Arabic (CA) forms. MSA is used in most Arab countries in different situations and places including on street signs, in newspapers, Television news, university, schools and books. When spontaneous speech samples are collected from participants, speakers use their own CA dialect. However, when a passage is read, participants use MSA because CA forms are not usually used in written texts (although CA is used in, for example, text messaging). Figure 2 summarizes the situations where MSA and CA are typically used.

Arabic has a complex morphology (38). It exhibits a discontinuous morphology that is based on the combination of the root and the word pattern (39). The root is exclusively made up of a sequence of consonants (usually three) that carry the core semantic information. The word pattern specifies the phonological structure and the morphosyntactic properties of the vowels, prefixes and suffixes that are then attached to the root to derive lexical meanings. In Arabic and in other Semitic languages, roots and word patterns are intertwined to form words across different lexical categories (e.g., nouns, verbs and adjectives). KTB is an example of a root that can be used to show how it implicates the meanings of the concept “write” (40). Table 1 gives seven examples of distinct word forms derived from this root. The examples are from different lexical categories and involve different vowels and prefixes.

**Table 1:** Words derived from the /ktb/ root.

**Word**	**Meaning**
Kataba	He wrote
Katabat	She wrote
Kitaab	Book
Maktaba	Library; bookstore
Kaatib	Writer
Kutayyib	Booklet
Maktuub	Written

## The Present Study

This study has two main goals. First, outlining a set of AEN_NWR stimuli that adheres to Arabic and English phonotactic constraints. Second, obtaining preliminary data on the effectiveness of the AEN_NWR in identifying stuttering in samples of AWS and CWS who speak Arabic as their first language. Specifically, we examined differences between CWS and AWS on accuracy of repeating non-words as the length of the non-words measured in syllables increased. It was expected that accuracy would decrease as non-words increased in syllable length for both groups; provided that non-words are of medium difficulty and thus ceiling and floor effects do not operate. Moreover, to provide support that the AEN_NWR is a reliable measure of stuttering, the empirical study reported here tested for a relation between AEN_NWR scores and the percentage of stuttered syllables (%SS). As advised in Riley's (41) manual, each stuttering instance was considered a single syllable and the %SS was obtained according to the manual. It was hypothesized that AEN_NWR scores would correlate with %SS in PWS, such that a lower AEN_NWR score would be associated with a higher %SS indicating higher levels of stuttering. Thus, results of stuttering assessment using a symptom-based procedure would be validated against AEN_NWR scores. Ideally, this preliminary testing should lead to further investigations of the AEN_NWR test items; and allow further refinement of the test until it is established as a sensitive measure that can equally assess Arabic- and English-speaking children.

## Materials and Methods

### Participants

Ten CWS and fourteen AWS participated. Information on participants' age, gender and stuttering severity are given in Table 2. All participants had been previously evaluated by Speech and language pathologist (SLPs) as exhibiting stuttering behaviors. Participants spoke Arabic as their first language. None of the participants had unusual phonological processes that affected syllable structure. Also, none of the participants had neurological deficits. Demographics on the children's age, gender and the dialect of Arabic spoken were collected from children's parents and obtained directly from the older groups before the experiment started. Participants received reimbursement for participation. Ethics approval was granted by UCL's Institutional Review Board number 0078/006.

**Table 2:** Participants in the study.

	**Group**	**Gender**	**Age**	**Dialect**	**Treatment History**	**%SS**	**AEN NWR**
1	Group 1	Male	6.5	Hijazi	Current	11	0
2		Male	9	Hijazi	Current	2	8
3		Male	9.0	Hijazi	Previous	2	13
4		Male	14	Kuwait	Previous	7	9
5		Male	11	Hijazi	Current	1.5	12
6		Male	11.5	Najdi	Current	6	9
7		Male	11.5	Gulf	Previous	3.5	6
8		Male	15.10	Omani	No treatment	11.5	0
9		Male	16.0	Egypt	Current	3	11
10		Male	16.7	Najdi	Current	0.8	21
1	Group 2	Female	19.2	Najdi	Previous	2.5	19
2		Female	20.1	Hijazi	Previous	7.5	3
3		Female	20.4	Najdi	No treatment	3.5	19
4		Male	22.0	Hijazi	Current	6.5	4
5		Female	23.1	Najdi	Previous	1	18
6		Female	24.12	Najdi	Previous	3	6
7		Female	24.8	Hijazi	Previous	7.5	2
8		Male	25	Najdi	No treatment	4	14
9		Female	25.1	Hijazi	Previous	6	1
10		Female	26.1	Najdi	Current	0.5	18
11		Male	29.4	Najdi	Previous	6.5	18
12		Female	31.0	Bahraini	No treatment	5	17
13		Male	21.9	Hijazi	Current	8	19
14		Male	21.9	Hijazi	Current	9	19

### Stimuli: Considerations for Designing Arabic English NWR Stimuli

This section explains the steps taken to generate the AEN_NWR stimuli.

#### Step 1

Overlapping phonotactic constraints across English and Arabic were identified to create the non-word candidates. For each syllable template, all possible phone sequences were created according to the following constraints.

#### Syllable Patterns

Two syllable templates were selected that are permitted in both languages.

1.CV–A short open syllable (Consonant–vowel)

2.CVC–A medium closed open syllable (Consonant–vowel–consonant)

A final dull syllable was also employed word-finally alone to generate word-final C.C clusters. Since the second segment of the dull syllable occupies an onset and a syllable onset must be supported by a nucleus, that onset then must be followed by an empty nucleus, hence the name dull syllable (42).

In Arabic, the syllable is always initiated with a single consonant, which requires an obligatory onset. To emphasize, no word can start with a vowel in Arabic. The maximum number of consonants allowed in the onset position is one in MSA and in most CA dialects. In English, the “C” in the onset is optional (an example where the “C” is absent in the word “eye”). However, there is a strong preference in English for a syllable to begin with a consonant as zero onset syllables (Ø) are rare (43). Thus, all syllables generated are well-formed with one C in the onset. The selected templates were strung together to form polysyllabic non-words that systematically increased in phonological difficulty.

Word-initial clusters were not allowed (they are permitted in English but not Arabic dialects). For example, Hijazi Arabic, which is spoken in the west region of Saudi Arabia precludes word-onset clusters. The non-words were generated to be appropriate for a range of dialects of Arabic, so they are suitable for use with participants from diverse geographical and socioeconomic backgrounds. Therefore, care was taken that the phonotactic constraints were not specific to one Arabic dialect because speakers of any dialect are potential users of AEN_NWR. Sequences of more than two consonants do not occur in any syllable position in Arabic and a vowel is obligatory within a syllable except for a final dull syllable. Because of the conflicting points of view about the existence of a coda in Arabic, and about the phonotactic restrictions on the consonant clusters that appear word-finally (whether they constitute a coda, or they are just adjacent consonants that appear word-finally), only the phonotactic constraints that the cluster should adhere to are discussed.

All possible phone sequences were created for each syllable template, with the following additional constraints:

#### Consonants and Vowels Selection

To be included in the AEN-NWR, a consonant or vowel had to occur as a phoneme or an allophone in both languages. The specific consonants and vowels that were selected were as follows:

##### Consonants

Consonant phonemes that exist in English and Arabic are the following: /f/ , /b/, /d/, /m/, /n/, /s/, /z/, /k/, /g/, / ∫/, /θ/ , /t/ /r/ and /l/ . The remaining consonant phonemes that exist in English or Arabic were not selected when the consonant phoneme does not have a corresponding phoneme in the other language. There are no corresponding phoneme for /t^ʕ^/, /d^ʕ^/ **/**ð^ʕ^**/**, /s^ʕ^/, /x/, /ɤ/ and /h/ in English. Also, there are no corresponding consonant phoneme for /p/, /v/ and /tʃ/ in Arabic. The consonant phoneme /t/ was selected although it is realized differently in the two languages. In Arabic, /t/ is dental involving simultaneous contact with the upper front teeth and the tongue tip and stopping the oral passage of air. In English, however, it is alveolar alone except when it occurs before dental fricatives (e.g., in words like “eighth” or between words like “at that”) when it is dental. These are sub-phonemic differences, and the phoneme category is used in the NWR test, as it is one of the phonemes that has an early age of acquisition.

###### Consonant Acquisition.

The age of acquisition of Arabic phonemes is generally similar to the corresponding ones in English, for common consonants although some allophonic variations between the two have been noted (44). In the latter study, a consonant was considered to have been acquired when at least 75% of children tested in each age group produced a consonant phoneme within a single word correctly in all positions of the word; initially, medially and finally. A Jordanian-Arabic-speaking child acquires /b/, /d/, /k/, /f/, /m/, /n/, /l/, /w/ in early childhood (2:0 to 3:10) and /s/, /h/ and /∫/ in later childhood (4:0 to 6:4) (44). Other consonants like /θ/ and /z/ are acquired later in Arabic (after 6:4) than in English. The acquisition criteria for English in the present study were adapted from Sander (45). According to Sander, children acquire /m/, /n/, /f/ and /w/ before the age of 3. The consonant phonemes /s/ and /∫/ are acquired in later childhood at the age of 4.5. Table 3 shows the age of acquisition for each of the selected consonant phonemes in both Arabic and English as reported in the studies that were mentioned above. It is important to note that these are average age estimates and the upper age limit in Sander (45) stops at an age level at which 90% of children are customarily producing the consonant phoneme. Information on this table were taken into account when designing non-words where consonants that are acquired earlier constituted most of the stimuli created making it possible for young children to produce them.

**Table 3:** Age of acquisition in Arabic and English.

**Consonant phoneme**	**Age of consonant acquisition in Arabic (Amayreh & Dyson, 1998) (44)**	**Age of consonant acquisition in English (Sander, 1972) (45)**.
/f/	(< 2;0 to 3;10)	(2;5 to 4;0)
/b/	(< 2;0 to 3;10)	(< 2;0 to 3;0)
/d/	(< 2;0 to 3;10)	(2;0 to 4;0)
/m/	(< 2;0 to 3;10)	(< 2;0 to 3;0)
/n/	(< 2;0 to 3;10)	(< 2;0 to 3;0)
/s/	(4;0 to 6;4)	(3;0 to 8;0)
/z/	(After 6;4)	(3;5 to 8;0)
/k/	(< 2;0 to 3;10)	(2;0 to 4;0)
/θ/	(After 6;4)	(4;5 to 7;0)
/ð/	(After 6;4)	(5;0 to 8;0)
/t/	(< 2;6 to 3;10)	(2;6 to 6;0)

##### Vowels

Three short vowels were selected [i, a, u] because each has an equivalent, or a near equivalent, that can be mapped across languages as shown in Table 4. Thus, a speaker of either of the two languages is not expected to have difficulty perceiving and then producing those vowels. Long vowels, however, were excluded because of differences between the two languages that may result in the speaker having difficulty perceiving contrasting forms because of the absence of some vowels from the participant's first language. Vowels in Arabic vary little among speakers of different Arabic dialects which employ the three short vowels that were listed above and another three long vowels. There are cases where long vowels might be analyzed as a sequence of two nuclei rather than a single branching nucleus (46). English has a larger number of vowels that do not have an equivalent in Arabic and thus may be problematic for an Arabic speaker to identify and produce. The quality of English long vowels also varies considerably between English accents. AlShanqiti (47) investigated how Saudi Arabic learners of English perceive and produce English vowels. She examined the problematic phonemic contrasts for learners of British English. The results showed that vowels that do not have counterparts in Arabic were more challenging for Arabic listeners to recognize. Also, Shafiro et al. (48) investigated the perception of American English vowels and consonants by native Arab speakers and Arab-English bilinguals. Vowel perception was less accurate than consonant perception in both groups. The authors attributed low accuracy in perceiving vowels to the bilingual participants' mapping of the larger Arabic English inventories to the smaller inventories of Arabic vowels. Moreover, a speaker might substitute one phoneme with another according to the speaker's first language. For example, in an Arabic word such as /ka:n/ (where the vowel is front low unrounded and long), English speakers would be expected to assimilate to the nearest vowel, which is the back low unrounded vowel [α] such as in the word /calm/ as reported in a study by Huthaily (35) where the difficulties in producing vowels for English learners of Arabic were examined. Huthaliy presented participants with the Arabic word [ra:tib] where the long vowel is front unrounded and long. All participants substituted the long vowel with [α] and used [α] to substitute for [a:] whenever it occurred. Since long vowels are commonly substituted, long vowels were excluded. For AEN_NWR, short vowels were included because they only differ in narrow phonetic details that should not be problematic for children or adults to recognize when listening to stimuli.

**Table 4:** Short vowels mapping across Arabic and English.

**Arabic short vowels**	**English short vowels**
/ı/ Front high unrounded short	/ı/ Front high unrounded lax
/℧/ Back high unrounded short	/℧/ Back high unrounded lax
/a/ Central low unrounded short	/æ/ Front low unrounded

#### Phonotactic Constraints

The following phonotactic constraints in both languages were implemented to ensure that the non-words that were created abide by the constraints of both languages.

##### Coda-Onset Clusters

“Coda” refers to a word-internal consonant, not a word-final consonant. This applies when using a syllable template with a coda like CVC for creating a non-word with two syllables or more, for example: CVC.CV, CVC.CVC. Word-internal codas are restricted by the following six constraints: (1) A coda must be a sonorant /m/,/n/,/w/, /r/,/l/ or an obstruent /k/ or fricative /s/, /f/ ; (2) A post-coda onset must be a plosive /t/, /k/, /d/, /g/, /b/; (3) No geminates are allowed as they are not allowed in English; (4) A nasal must be homorganic with a following onset: [mb, nt, nd]; (5) [s] can only appear before a plosive voiceless onset: [st, sk, sf]; and (6) [r] can appear word-finally in General American English but not in non-rhotic accents such as British English hence its usage is restricted to onset positions alone. All of the clusters allowed by these restrictions are shared by Arabic and English.

##### Word-Final Clusters

As mentioned above, the phonotactics of the word-final consonants in English are similar to those of the internal coda. In other words, a word-final cluster behaves like an internal C.C because it is also a coda onset cluster. Consequently, because of the parallelism between the two domains, the phonotactic constraints need to be stated once only (42). Generally, the two phonemes in the word-final consonant cluster in English must conform to the sonority sequencing phonotactic principle (SSP). SSP was first introduced by (49) and it aimed to characterize the syllable structure in terms of sonority. Thus, in a C.C coda cluster, the first coda consonant should be higher in sonority relative to the second coda consonant. There are exceptions when word-final pairs of consonants violate the SSP. These occur in a sequence of two stops that are not homorganic (e.g., act), and a sequence of a stop + /s/ (e.g., lapse and tax) (50). Moreover, there are final sequences that show no evidence of phonotactic constraints which are usually generated by suffixation. In Arabic, however, the SSP is not a reliable predictor of the sequence of two consonant phonemes that occur word-finally. There are many Arab words, in MSA and in several other Arabic dialects that violate the SSP. AlTamini and AlShboul (51) conducted a study of MSA coda cluster phonotactics to assess applicability of SSP. They assumed that any consonant that follows the last vowel of a word belongs to a coda. The authors used a sample of around 500 CVCC lexical items that were collected from The Hans Wehr Dictionary of Modern Written Arabic. It is important to note here that looking at the words that were selected from the dictionary, many of them are nouns of high frequency and they are usually pronounced the same way in MSA and Arabic dialects as in /^ʕ^aks/ “reverse”, /s'ubh/ “morning”, /wadJh/ “face”. The results showed that contrary to what is widely reported in the literature on the compliance of the CC coda with SSP, this was only true for 42% of the cases. The remaining 58% of the cases violated the SSP sonority hierarchy as follows: (1) reversal (49% of the C.C coda clusters showed a rise in sonority in which the coda first consonant had lower sonority relative to the coda second consonant); or (2) plateau in which the C.C cluster consonants are of almost equal sonority. The results challenge the fact that the phonotactics of the Arabic C.C coda are sonority based because SSP was violated in more than 50% of cases. The authors raised the idea of re-considering a more theoretical model outside the scope of SSP that has been long thought of as governing complex coda syllables.

#### Step 2

Non-word candidates were selected for each syllable length as follows: First, all permitted syllable combinations which could serve as templates were created for each syllable length; 100 of these templates per syllable length were then selected at random. Next, consonant and vowel phones were selected at random, apart from those barred by the constraints listed above (i.e. consonants and vowel selection). The selected phones were entered into the template for each syllable length. Finally, individual syllables were combined. Given all the constraints above, the AEN_NWR test should meet the goals of including segments that a speaker of Arabic or English can pronounce and do so in comparable ways across these languages provided that the speaker is equally fluent in both languages. In the study, even when phonologically complex materials for the two languages are tested, testing starts with “easy” materials with minimal use of clusters. Then, complexity is increased systematically in terms of the number of segments and syllable structures. Appendix 1 shows the orthographic transcription of the final two, three and four syllable AEN_NWR stimuli.

#### Step 3

Stress, as a linguistic phenomenon, occurs in both languages. The two languages are similar in terms of the association between stress and heavy syllables; Arabic and English are both quantity-sensitive languages where heavy syllables attract stress (52, 53). In order to meet the phonological requirement of testing across the two languages, language-specific stress contrasts were avoided by producing all syllables with equal stress, except for the CVCC heavy syllables which mark the end of some stimuli. Additionally, another set of identical non-word unstressed syllables was developed where short vowels were reduced to schwa; hence the vowels were homogenized (Note: this set was not used in the current experiment). This was done because there are differences in vowel quality and stress between Arabic and English. When a participant's repetition of an AEN_NWR stimulus was evaluated, any differences in produced stress patterns or vowel quality were ignored and only consonants were considered in scoring.

#### Step 4

Checks were made to ensure none of the phone sequences are words in either of the languages using Aralex (54). Aralex is a lexical database for MSA that provides token frequencies of roots and word patterns that integrates information from two sources: (1) a 40 million word corpus derived from different newspapers covering various topics such as politics, sport and culture; and (2) a dictionary compromised of 37,494 entries that provides information and token and type frequencies of Arabic words and morphemes. Although Aralex was created using MSA rather than a spoken dialect of Arabic, it still meets the requirements for a lexical database that can be checked for lexicality effects for the following reasons. (1) Despite the existence of many dialects across different Arabic countries, speakers of the language have a single inventory of phonemes. (2) MSA and spoken Arabic forms present with different phonological, syntactic and lexical systems where each fulfills distinct sociolinguistic functions. The database was built from MSA using text from newspapers but when dialectal words were found they were retained in the corpus but flagged as such (54). It is worth noting here that Arab dialects are rarely written and they are mostly used for speech communication alone. The Aralex database (2010) has a user-friendly interface that consists of 12 boxes where filled boxes can be used to input a search string. The user has the option of displaying the results in English or Arabic Unicode. For example, the orthographic form window takes as input an Arabic or English script and displays the selected results either in Arabic or English.

### Experimental Procedure

Participants were tested in a quiet setting in one session of approximately 15 min. The experimenter conducted two tasks: (1) elicitation of spontaneous speech samples and (2) administration of the AEN_NWR. The study used a within-participants design as participants completed both tasks; and the two tasks were conducted in randomized order. The complete session was recorded on a Sony DAT audio-recorder using a Sennheiser K6 microphone and audacity software. For the spontaneous speech, samples of 200 syllables from all participants were obtained. These were elicited during conversational speech with the researcher using topics of interest, such as school, travel, books and hobbies. When necessary, picture material from Riley (6) was also used to elicit speech from children. For the AEN_NWR, the 28 non-words listed in Appendix 1 were used as follows. All stimuli from the three sets (i.e., two syllables, three syllables and four syllables) were presented in standard order, in which stimulus length systematically increased. Participants were exposed to all stimuli regardless of their performance. Participants were informed that they would hear made-up words; i.e., ones that do not exist either in Arabic or English. The examiner then gave the following instructions to the participant: “I am going to play some made-up words to you through the headphones and I want you to repeat them as accurately as you can. You will have to listen carefully because you will only hear them once”. Participants were allowed as much time as was necessary to respond. There were two practice items to make sure the output volume was appropriate, and the participant understood the nature of the task. The non-words were pre-recorded to ensure that factors such as differences in word stress patterns and accent would not affect the results. Recording of materials the participants heard took place in an anechoic chamber and were obtained from a male professional phonetician who was phonetically trained in Arabic and English^2^.

### Data Processing, Scoring and Reliability

#### Spontaneous Speech

The audio recordings of the participants' speech were orthographically transcribed after replaying them as many times as necessary by the researcher. The percentage of stuttered syllables (%SS) from the first 200 syllables were obtained following guidelines from Riley (6). Todd et al. (55) confirmed that a 200-syllable long speech sample was sufficient to obtain a reliable SSI score. Guidelines from the original SSI-4 (6) were followed to score the first 200 syllables of all samples collected. %SS was calculated by determining the number of stuttered syllables and the total number of syllables. The number of stuttered syllables was then divided by the total number of syllables and multiplied by 100, resulting in the percentage of stuttered syllables %SS. A syllable was stuttered if the speaker exhibited one of the three disfluency characteristics: (1) repetition indicated by multiple repetition of a sound or a syllable that was not a word; (2) prolongation indicated by abnormal lengthening during the production of a phoneme; (3) break within a syllable.

#### AEN_NWR Scoring

The recorded non-words were scored offline at the whole-word level. The non-word phonemic response transcriptions and target transcriptions for each non-word were aligned to identify how the two transcriptions differed. A correct response occurred when all consonants in each non-word were pronounced correctly. Errors on consonant production included deletion, substitution or insertion.

Responses were scored as “incorrect” if they contained one or more consonant errors. Vowels were ignored during scoring because of the subtle differences between dialects. To ensure reliability, responses from 20% of the participants, selected randomly, were scored independently by a second trained phonetician. Agreement on the number of correct stimuli was 88%. Data from the first author was used in statistical analysis.

## Results

### Performance of CWS and AWS on the AEN_NWR

To review, we were interested in examining the effect of age group and non-word length on accuracy of repeating non-words. A repeated Measures ANOVA with the between-participants factor of Group (CWS vs. AWS) and a within-participants factor of Syllable Length (2-, 3, and 4-syllables), was conducted. The dependent variable was the mean % of correctly repeated non-words. Results revealed a main effect for Syllable Length F (2,44) =11.84, P <0.000), partial $\eta_{p}^{2}=$ 0.4. Overall, there was no significant between-participant difference F (1,22) = 3.1, p = 0.09, partial $\eta_{p}^{2}$ = 0.1.

**Figure 3:**
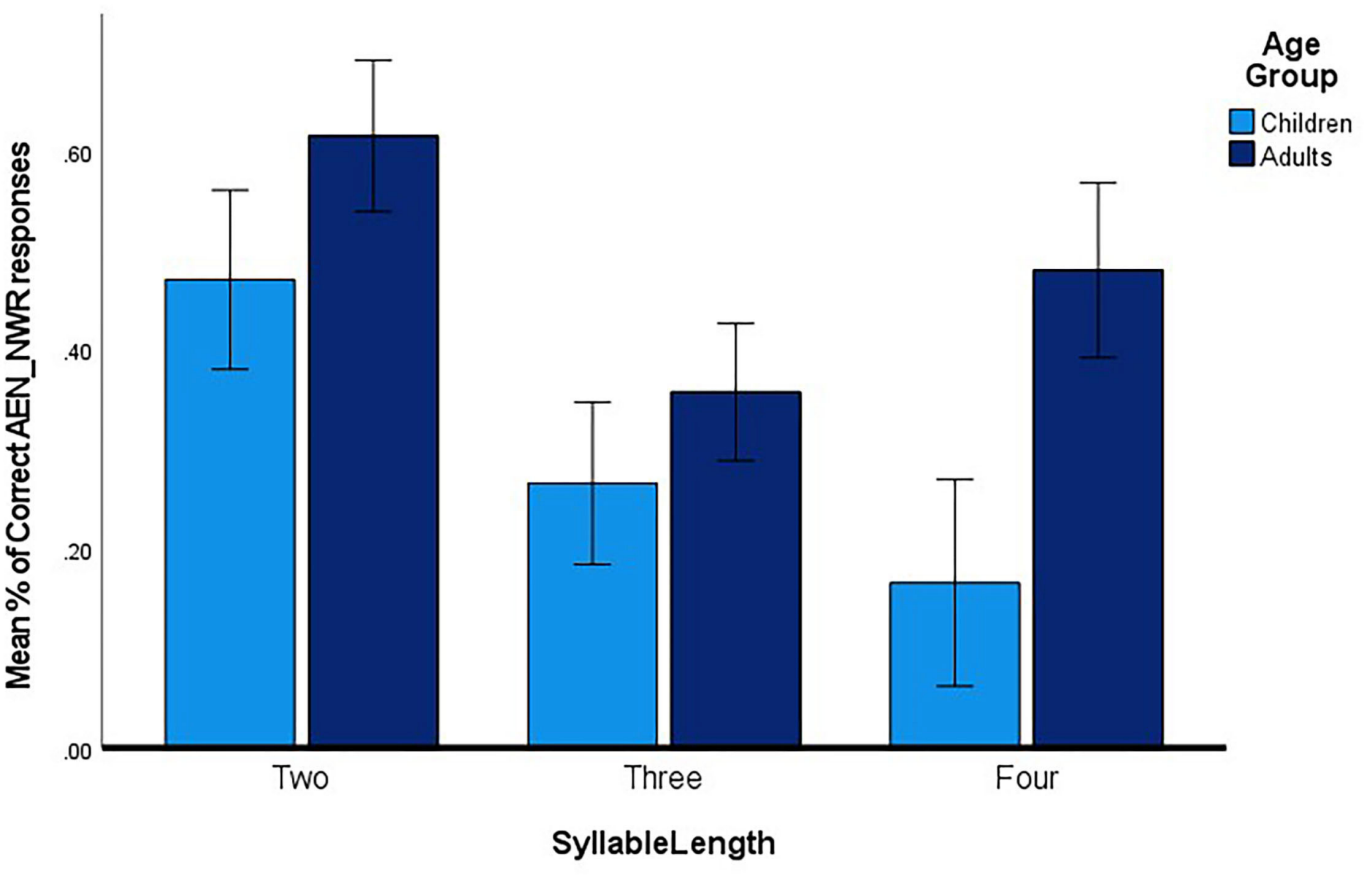
The mean % of accurate productions on the AEN_NWR for producing the non-word at each syllable length for CWS and AWS. Age group is indicated in the inset.

The differences between the CWS and AWS were inspected at each syllable length using independent samples *t*-tests. Significant difference between the two groups were found for non-words at the 4-syllable length only F (1,22) = 7.60. *p* = 0.01). No significant differences were noted at the 2-syllable, or 3-syllable lengths. Figure 3 summarizes the results.

### Validating AEN_NWR Against Symptom-Based Stuttering Measure

**Figure 4:**
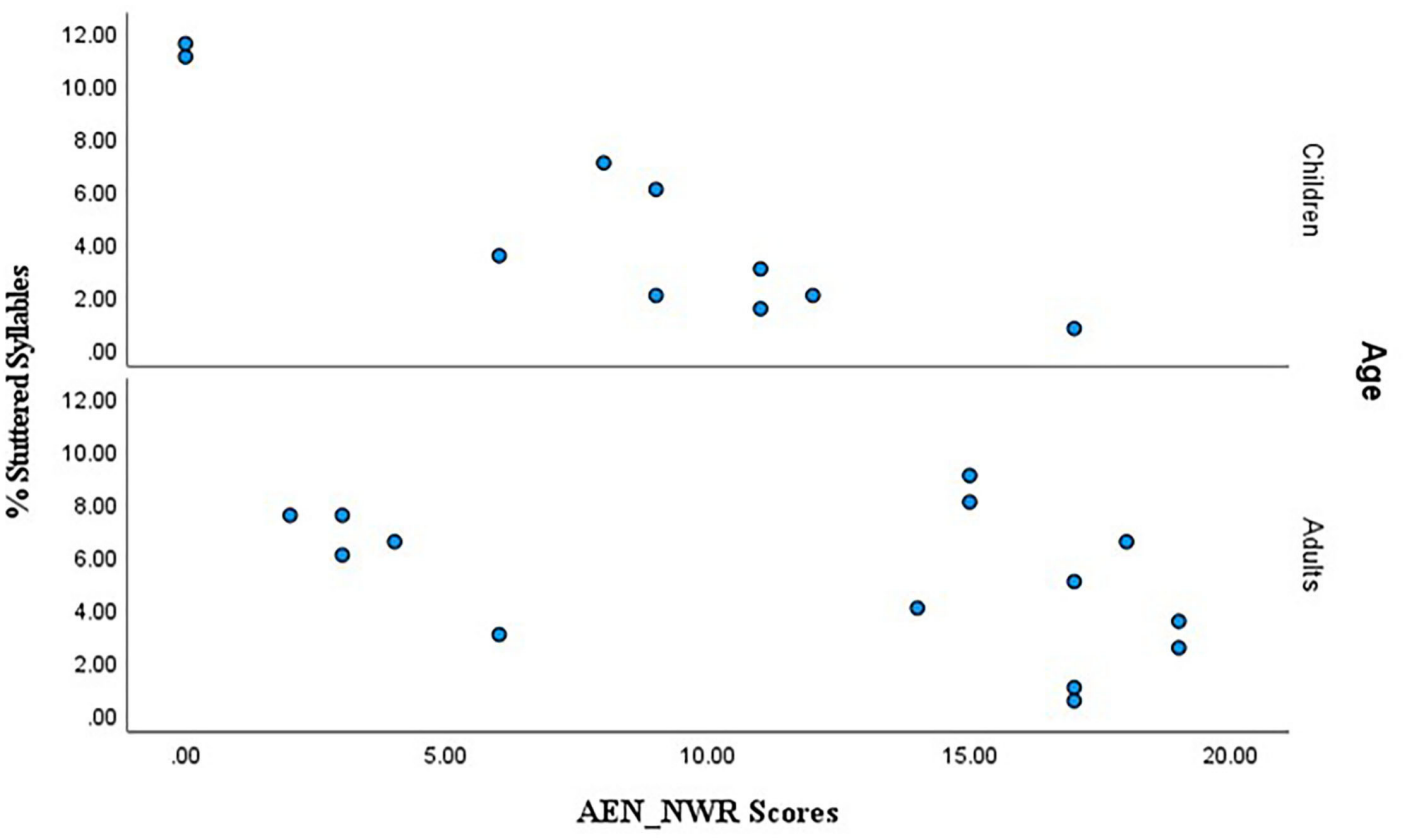
Scatter plot of raw %SS (frequency scores) (Y-axis) against total AEN_NWR scores (X-axis) with panels for CWS and AWS at top and bottom.

Simple linear regression was used to assess whether AEN_NWR scores predicted %SS scores. A correlation analysis using Pearson's r can be used since the variables were measured on a continuous scale. A significant negative correlation between the AEN_NWR and %SS scores was found using Pearson's product-moment correlation coefficient (r (25) = −0.5), *p* < 0.000), indicating that higher AEN_NWR scores were associated with lower %SS, making AEN_NWR scores a significant predictor of %SS. Performance of each group was inspected again in a separate analysis. A significant negative correlation between the AEN_NWR and %SS for CWS was found (r (10) = −0.9. *p* < 0.000). However, the correlation between AEN_NWR and %SS was not significant for AWS (*p* > 0.05). Figure 4 summarizes the relationship with separate panels for AWS and CWS.

## Discussion

The purpose of this paper was to describe how a set of stimuli for an Arabic-English non-word repetition (AEN_NWR) task were developed, and to examine preliminary data from a small clinical sample of Arabic-speaking CWS and AWS. The study was motivated by Howell et al.'s (9) finding that showed that the “Universal” NWR (UNWR) can identify stuttering in preschool children, irrespective of their first language. However, the UNWR does not apply to Semitic languages such as Arabic. This issue was addressed in this paper by constructing AEN_NWR items that obeyed the phonotactic constraints of Arabic and English. The test had three groups of items that varied in length between two and four syllables. Based on the literature on phonological working memory and stuttering, we expected that, performance on AEN_NWR should decrease as the number of syllables in the stimuli increased. Because performance on the NWR develops with age (23), we recruited clinical samples that comprised CWS and AWS to examine developmental changes. Finally, we validated overall AEN_NWR accuracy by comparing participants' scores against a standard symptom-based procedure, the percentage of stuttered syllables %SS.

### Accuracy in Repeating AEN_NWR Items

Regardless of age, non-word length affected accuracy of performance. This was demonstrated by the main effect of syllable length. The 2-syllable non-words were repeated more accurately than 3-syllable non-words, and 3-syllable non-words were repeated more accurately than 4-syllable non-words. These findings add evidence about the effect of phonological memory on individuals who stutter [e.g., (24, 34)]. As non-words increase in length, they take additional time to be perceived and repeated, and consequently their phonological representations may be prone to decay before they can be rehearsed and articulated (56).

As the AEN_NWR is a new measure, we performed separate analysis for CWS and AWS to examine the effect of age on accuracy of performance. Although AWS consistently performed better at all syllable lengths, significant differences were reached only at the 4-syllable length non-words. These findings could be interpreted as preliminary evidence that non-words of at least 4-syllable length are required for the AEN_NWR to be sensitive to age differences. However, it is too early to make such a generalization without increasing participant numbers in both groups. Having a large number of participants from a wide age range could also inform decisions such as: (1) how many items are required at each syllable length; and (2) at what syllable length do CWS and AWS show differences in performance. Ebert et al. (57) noted that non-word stimuli should include enough variants to capture the wide range of non-word repetition ability levels. Hence, the variation in average performance on the AEN_NWR across ages could inform design considerations of the task to make it suitable to test preschool children.

### Preliminary Validation of AEN_NWR

Evidence was provided that the AEN_NWR is a reliable measure of phonological skills and hence of fluency of speech. It was hypothesized that, overall, AEN_NWR scores should correlate negatively with %SS (i.e., percentages of stuttered syllables, %SS). This hypothesis was partially supported; it appears that participants who showed a higher percentage of stuttered syllables/dysfluent events scored lower on the AEN_NWR. These findings are consistent with the results by Howell et al. (9) who showed a relationship between %SS and UNWR scores. Thus, this provides support that NWR as an established measure of phonological skills for participants with stuttering symptoms. The correlation with the %SS could be interpreted as an indication that the test has good potential for identifying preschool children with speech disfluency. However, such a conclusion need qualifying until the current results are compared with results of control groups of adults who do not stutter and children who do not stutter. The importance of examining developmental differences between AWS and CWS is linked to the ceiling and floor effects, which are common to NWR tasks (25). Ceiling effects operate for non-words of short syllable lengths and floor effects operate for the longer ones, and this can limit the ability to detect individual differences Munson (58).

When the two age groups used in this study were examined separately, the correlation coefficient for CWS as a group was higher than that for the AWS. This finding could be attributed to the fact that some AWS had severe stuttering symptoms. That is, their mean %SS was 4.94; SD 3.1 and seven AWS scored above the mean. This potentially created a skewed sample, which in turn had an effect on the relationship with AEN_NWR. It is worth mentioning that many of the AWS had therapy after their teenage years but despite that their stuttering persisted, although they continued receiving speech therapy services to improve their fluency. Moreover, the fact that AWS showed better performance than CWS could be interpreted as an indication of floor effects operating, particularly for the longer syllables. In fact, ceiling and floor effects may be complicating factors for many studies on NWR accuracy (54). Also in the stuttering population, longer nonwords might pose an additional challenge for CWS relative to AWS. This is particularly true because scoring is done at the word level rather than phonemic level, another factor that could obscure individual differences in performance.

## Conclusion

The results indicated that the proposed non-word stimuli of the AEN_NWR were broadly appropriate for the stuttering population tested. Floor effects appeared to be influencing performance of CWS, but as mentioned, these are common in NWR tasks. The design consideration behind the AEN_NWR involved eliminating any influences that language history could have on an individual's performance. At this stage, only Arabic speakers were tested, but future work needs to evaluate the performance of English speakers and to compare the two groups (including people who are fluent or who stutter). The proposed set of stimuli conforms to accepted standards for NWR tasks including the following: language-specific phonotactic constraints (Arabic and English), avoiding later-developing consonants, and minimizing potential resemblance between real words and non-words. Furthermore, the test does not require knowledge of lexical semantics for either of the two languages. This aids in reducing any biases that speakers are relying on existing semantic knowledge from their stored language memory.

### Limitation and Future Direction

This paper makes a novel contribution to the subject area of stuttering as an aspect of language disorder, and to screening procedures of stuttering; particularly in the Arabic language, in which the literature is sparse. To the best of our knowledge, this is the first time that non-word repetition abilities have been investigated in Arabic speakers in stuttering research. While the current preliminary data provide promising results, there are some limitations that should be raised. First, all participants in this study come from a clinical sample and had been diagnosed as displaying stuttering symptoms; However, different SLPs may use different guidelines to diagnose stuttering due to the lack of norms for assessing fluency in Arabic. The speech samples obtained in this study were in Arabic as the aim was to develop and standardize an instrument to assess fluency for Arabic preschool children; however for analysis of speech symptoms, the guidelines of SSI were followed with respect to what is counted as a stuttering symptom and SSI was developed for English PWS. Although a strong correlation was found between the %SS and the AEN_NWR, which might suggest that the guidelines can be generalized to Arabic, having guidelines designed specifically to Arabic could result in even a stronger correlations. To explain in more detail, the data used to evaluate SSI statistically were collected from English speakers who stuttered, and it used English passages that had to be read as well as others that were spoken spontaneously. Consequently, the norms do not apply to the Arabic version as the standardization has not been conducted. Moreover, the test is not appropriate for assessment of Arabic in terms of the procedures used in SSI for counting the number of production units (i.e. syllables for English) as well as the specifications of stuttered events. Thus, in parallel to standardizing the AEN_NWR, work is ongoing to establish clear guidelines for assessing spontaneous speech samples in Arabic. This includes instructions on syllables counts and disfluency counts. Second, consistency of the results should be examined by test-retest reliability, which is a necessary measure for an NWR to be considered reliable for diagnostic purposes (59). Third, probably the biggest limitation of this study concerns the number of participants. Although the sample size is reasonable compared to other studies that examined the non-word repetition abilities in PWS [e.g., Hakim and Ratner (24) had eight CWS and Sugathan and Maruthy (28) had 17 CWS], it is important to replicate the results of this preliminary study using larger number of participants. This is particularly the case because participants in this study were from different age groups and there may be developmental differences between them. Having more participants could also permit assessing phonological performance at every non-word length to examine the relationship between repetition accuracy and phonological complexity.

Finally, it would be of interest to examine matched groups of: (1) controls of Arabic speakers who do not stutter; (2) English speakers who stutter and examine their performance on the AEN_NWR. Arabic speakers of dialects other than Saudi could also be examined as additional comparison cohort; this would allow investigating the influence of dialect on AEN_NWR performance.

## Data Availability Statement

The raw data supporting the conclusions of this article will be made available by the authors, without undue reservation.

## Ethics Statement

The studies involving human participants were reviewed and approved by UCL Ethics Research Committee. Written informed consent to participate in this study was provided by the participants' legal guardian/next of kin.

## Author Contributions

PH and RA designed the study, performed the research, analyzed the data, and wrote the manuscript. PH, JH, and RA developed the non-word stimuli. SB assisted in recruiting participants. All authors contributed to the article and approved the submitted version.

## Funding

The authors extend their appreciation to the King Salman Centre for Disability Research for funding this work through Research Group No. KSRG-2022-001.

## Conflict of Interest

The authors declare that the research was conducted in the absence of any commercial or financial relationships that could be construed as a potential conflict of interest.

## Correction Note

22 July 2024 A correction has been made to this article. Details can be found at: 10.3389/fped.2024.1280806.

7 July 2025 A correction has been made to this article. Details can be found at: 10.3389/fped.2025.1652470.

## Publisher's Note

All claims expressed in this article are solely those of the authors and do not necessarily represent those of their affiliated organizations, or those of the publisher, the editors and the reviewers. Any product that may be evaluated in this article, or claim that may be made by its manufacturer, is not guaranteed or endorsed by the publisher.

## Footnotes

^1^The 20 languages the UNWR applies to are English, Polish, Romanian, European Portuguese, Bulgarian, Serbo-Croat-Bosnian, Czech, Dutch, French, German, Hungarian, Slovene, Swedish, Danish, Norwegian, Russian, Latvian, Ukrainian, Urdu-Hindi and Bengali.

^2^The authors would like to thank Christopher Lucas, SOAS University of London, for recording the non-word stimuli. The recorded materials can be.

## Appendix

**Table A1:** Orthographic transcription of the AEN_NWR stimuli.

**Two syllable**	**Three syllable**	**Four syllable**
Sibad	Dabibum	Lisakubam
Damif	Sifakuf	Zimtakazum
Fibil	Natadulb	Rifatanult
Manib	Sigadilk	Dakanufast
Tundan	Lazafusk	Kabalikift
Nastim	Ristudab	
Bundaf	Mundatis	
Nambik	Randitak	
Saftif	Luntambilf	
Takisk	Rimbadusk	
Bamift		
