# Corrigendum: Identifying stuttering in Arabic speakers who stutter: development of a non-word repetition task and preliminary results

**DOI:** 10.3389/fped.2024.1280806

**Published:** 2024-07-22

**Authors:** Roaa Alsulaiman, John Harris, Sarah Bamaas, Peter Howell

**Affiliations:** ^1^ University College London, London, United Kingdom; ^2^ Jeddah Center for Speech, Hearing and Medical Rehabilitation, Jeddah, Saudi Arabia

**Keywords:** fluency, developmental stuttering, screening, Arabic, speech disfluency, word-finding

A Corrigendum on Identifying stuttering in Arabic speakers who stutter: development of a non-word repetition task and preliminary results By Alsulaiman R, Harris J, Bamaas S and Howell P (2022). Front. Pediatr. 10:750126. doi: 10.3389/fped.2022.750126

Upon request of the author of reference 46, the data and the references included in Table 3 have been updated after removing the citation ‘Bahakeem, M; (2016) Phonological Development in Saudi-Arabic-Speaking Children. Unpublished Doctoral thesis, UCL (University College London)’. This change does not affect the original conclusion of the article. The revised Table 3 and its caption appear below.

**Table 3:** Age of acquisition in Arabic and English.

Consonant Phoneme	Age of consonant acquisition in Arabic (Amayreh & Dyson, 1998) (44)	Age of consonant acquisition in English (Sander, 1972) (45)
/f/	(<2;0–3;10)	(2;5–4;0)
/b/	(<2;0–3;10)	(<2;0–3;0)
/d/	(<2;0–3;10)	(2;0–4;0)
/m/	(<2;0–3;10)	(<2;0–3;0)
/n/	(<2;0–3;10)	(<2;0–3;0)
/s/	(4;0–6;4)	(3;0–8;0)
/z/	(After 6;4)	(3;5–8;0)
/k/	(<2;0–3;10)	(2;0–4;0)
/*θ*/	(After 6;4)	(4;5–7;0)
/ð/	(After 6;4)	(5;0–8;0)
/t/	(<2;6–3;10)	(2;6–6;0)

As such, reference 46 “Bahakeem M. Phonological Development In: Saudi-Arabic-Speaking Children. Unpublished Doctoral thesis. UCL (University College London). (2016)” has been deleted from the references list.

This does not change the scientific conclusions of the article in any way. The original article has been updated.

