# Correction: Identifying stuttering in Arabic speakers who stutter: development of a non-word repetition task and preliminary results

**DOI:** 10.3389/fped.2025.1652470

**Published:** 2025-07-07

**Authors:** Frontiers Production Office

**Affiliations:** Frontiers Media SA, Lausanne, Switzerland

**Keywords:** fluency, stuttering, screening, Arabic, speech disfluency, word-finding, non-word, diversity

**A Correction on**
Identifying stuttering in Arabic speakers who stutter: development of a non-word repetition task and preliminary results By Alsulaiman R, Harris J, Bamaas S and Howell P (2022). Front. Pediatr. 10:750126. doi: 10.3389/fped.2022.750126

A correction has been made to the section **Materials and Methods**, Stimuli: Considerations for Designing Arabic English NWR Stimuli, Consonants and Vowels Selection, Consonants, paragraph two. The sentence was corrected to be more specific.

The sentence previously stated:

“An Arabic-speaking child acquires /b/, /d/, /k/, /f/, /m/, /n/, /l/, /w/ in early childhood (2:0 to 3:10) and /s/, /h/ and /∫/ in later childhood (4:0 to 6:4) (44).”

The corrected sentence appears below:

“A Jordanian-Arabic-speaking child acquires /b/, /d/, /k/, /f/, /m/, /n/, /l/, /w/ in early childhood (2:0 to 3:10) and /s/, /h/ and /∫/ in later childhood (4:0 to 6:4) (44).”

The original version of this article has been updated.

